# Evaluating the Small-World-Ness of a Sampled Network: Functional Connectivity of Entorhinal-Hippocampal Circuitry

**DOI:** 10.1038/srep21468

**Published:** 2016-02-23

**Authors:** Qi She, Guanrong Chen, Rosa H. M. Chan

**Affiliations:** 1 City University of Hong Kong, the Department of Electronic Engineering, Hong Kong

## Abstract

The amount of publicly accessible experimental data has gradually increased in recent years, which makes it possible to reconsider many longstanding questions in neuroscience. In this paper, an efficient framework is presented for reconstructing functional connectivity using experimental spike-train data. A modified generalized linear model (*GLM*) with L1-norm penalty was used to investigate 10 datasets. These datasets contain spike-train data collected from the entorhinal-hippocampal region in the brains of rats performing different tasks. The analysis shows that entorhinal-hippocampal network of well-trained rats demonstrated significant small-world features. It is found that the connectivity structure generated by distance-dependent models is responsible for the observed small-world features of the reconstructed networks. The models are utilized to simulate a subset of units recorded from a large biological neural network using multiple electrodes. Two metrics for quantifying the small-world-ness both suggest that the reconstructed network from the sampled nodes estimates a more prominent small-world-ness feature than that of the original unknown network when the number of recorded neurons is small. Finally, this study shows that it is feasible to adjust the estimated small-world-ness results based on the number of neurons recorded to provide a more accurate reference of the network property.

Developing mathematical models to describe how a mammalian nervous system functions is crucial to neuroscience, medicine, and bio-engineering. However, bridges between existing statistical studies of behaviors and large-scale mechanistic modeling initiatives have yet to be built. Recently, a number of high-quality electrophysiological recording databases, such as the Collaborative Research in Computational Neuroscience (*CRCNS*) program^1^, have been made publicly accessible for computational neuroscience research. Yet, there do not seem to be comparative studies which investigate the neural dynamics and the underlying network topology in different parts of the nervous system by examining the recordings published in these databases within a general modeling framework. The approach taken in the present study is to begin with deriving a data-driven input-output model and its corresponding network topology of a brain sub-region from the electrophysiological data, before proceeding to look into behavior.

Studies have identified small-world features - short path lengths and large clustering coefficients in biological neural networks^2^. However, the current noninvasive neural imaging techniques are unable to capture the whole brain’s activity on a neuronal level, particularly for the case of functional connectivity^3^. Functional connectivity can be well quantified based on statistical dependencies, such as transfer entropy, coherence, and correlations. It incorporates the information from the structural connectivity, which is developed from axon and dendrite formation, and contributes to the region specific local circuitry functions of the brain^4^. To investigate the brain activities on the neuronal level, only limited portions of brain tissues could be analyzed even using the best available invasive techniques^5^,^6^. Such techniques, e.g. multi-electrodes commonly used in recording electrophysiology, are sampling only some of the neurons from a large regional network. Therefore, the resulting network topology is only a sub-graph of the original network. Previous studies have focused on how to derive representative samples from large real networks^7^,^8^, this is because some properties such as average path lengths cannot be found efficiently from the huge graphs. Yet, there is a lack of complex network studies investigating whether the properties derived from a sampled network can represent the original network accurately on the neuronal level. Hence, this paper investigates how the multi-electrode sampling method used in experiments can influence the small-world features obtained. To compare the small-world features between the original network (*ON*) and the sampled network (*SN*), a quantitative method describing the small-world-ness has to be identified. It needs to be validated for an objective comparison of significance in small-world-ness based on path lengths and clustering coefficients. Performance evaluation of the new metrics through a simulated distance-dependent probability model, which describes the multi-electrode sampling method^9^, is therefore crucial.

This paper presents perhaps the first comparative study of the functional network topology, analyzing spike-train datasets collected from rats performing various different tasks. The study focuses on the entorhinal-hippocampal area, an important brain region for learning and memory^1^. The graph of network connectivity was constructed in two steps. First, we characterized the local neural circuits from multiple time series spike trains using a generalized linear model (*GLM*) with regularization terms, instead of merely pairwise measures^10^. System dynamics were captured by the kernels reconstructed from the weighted sum of Lageurre basis functions. Such a technique also significantly reduces the number of parameters to be estimated. Second, after the graph of the connectivity was estimated, we analyzed its structural properties using graph theory. The resulting graph of the entorhinal-hippocampal region demonstrated small-world properties. We then evaluated the performance of two metrics for quantifying the small-world-ness of the sampled network and simulated a large network using the distance-dependent probability model. We found that we overestimated the small-world-ness using the multi-electrode method when the number of sampled neurons was smaller than 100. Below 100 the first metric, *S*_*w*_, performs unstably while the second metric, 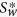, shows consistent results - overestimation. Finally, based on the topology of the recorded neurons, we evaluated the small-world-ness of the functional network. For a more precise reference of the network property, we developed a means to adjust the small-world-ness measure based on the number of sampled neurons. In addition, as the ability to control the reconstructed network is critical to guide the dynamics of the whole network, we have also studied network reconstructed from real datasets to find the “key” nodes to structurally control the entire system.

## Results

### Model Estimation

**Figure 1:**
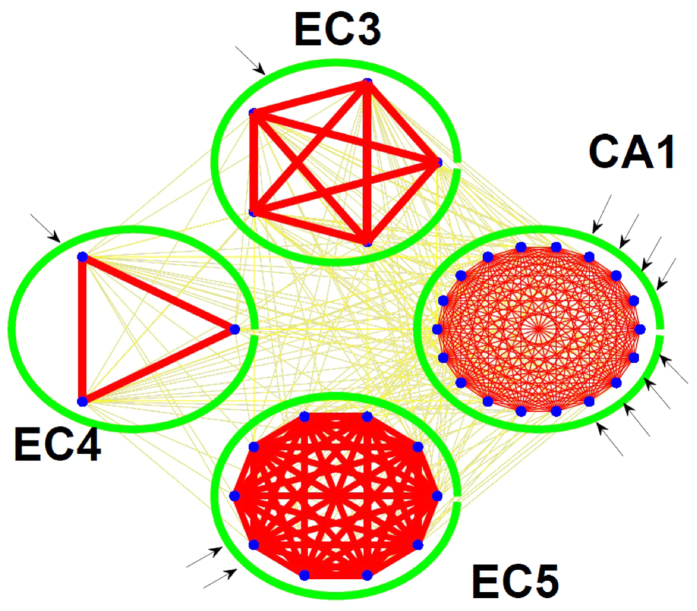
Reconstructed Cellular Functional Network. The arrows correspond to the number of electrodes inserted into CA1, EC3, EC4, EC5 regions. Each region is highlighted by a green circle. Blue dots represent neurons, and red lines indicate the connections between neurons within a region. Yellow lines highlight the connections between regions.

This study investigates the relationship among neurons with firing rates higher than 0.5 Hz. The K-S plot and the prediction accuracy provide us with parameters for model verification. The parameters selected as significant were used to reconstruct the neural network model. Figure 1 presents the reconstructed functional network from the *Rat #3* (*ec016*) recorded for 28 minutes in session *437* (*ec016.437*). Before the recording, the rat had trained on the wheel task 8 times. Our reconstructed functional network contains 36 neurons across 4 regions in entorhinal-hippocampual area and, in each region, the neurons are nearly fully connected, which shows a strong regional small-world feature. Although the structural (anatomical) connectivity at hippocampus is relatively simpler than most of the other brain regions, the functional connectivity of entorhinal-hippocampual network is still sophisticated^12^. The functional connectivity that we derived from the experimental datasets reflected the actual relationships between neural activities at different regions of entorhinal cortex (EC) and hippocampal CA1 region.

### EC and CA1 Form a Significant Small-world Network

Data collected from different rats performing various experimental tasks were used to reconstruct the corresponding functional connectivity graphs. In Table 1, the true positive (*TP*) is the accuracy rate of predicting the spike train having a spike. The true negative (*TN*) corresponds to the accuracy for having no spike. We use Receiver Operating Characteristic (*ROC*) curve to determine the threshold to get the highest *TP* and *TN*. Table 1 shows that the reconstructed network has a small average path length and a very high average clustering coefficient (‘1’ means that neighbors are fully connected). Both suggest that the entorhinal-hippocampal regions studied are small-world networks. Also, the *Rat #2* in *ec013.578* session underwent more training sessions than *Rat #3* in *ec016.437*, which shows more significant small-world features in this network. As shown in the last 3 rows in Table 1, the more training *Rat #4* had in Bigsquare task, the more connections established in the final reconstructed network.

### Validation Using K-S Plot

**Figure 2:**
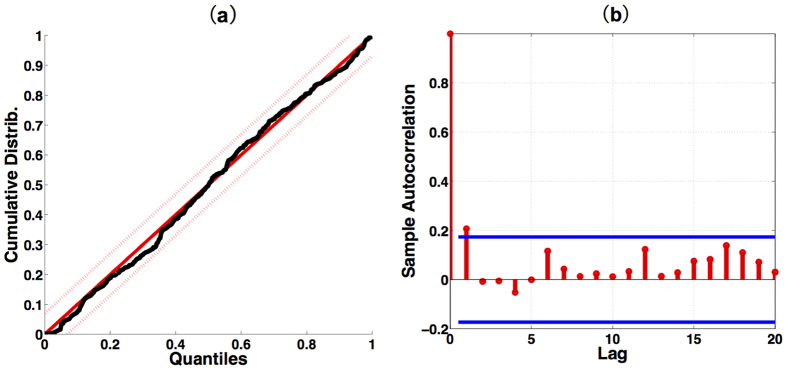
(**a**) K-S plot of one example neuron from session *ec012ec.187*. Straight dashed lines in K-S plot are the 95% confidence bounds; (**b**) Autocorrelation plot of subsequent transformed values. Blue lines are the 95% confidence bounds without correlation.

The models are statistically validated. An example K-S plot is shown in Figure 2. The K-S plot (*solid*) line lies within the dashed horizontal lines with 95% confidence. The second column shows insignificant autocorrelation between the transformed rescaled times. Both suggest that the estimated model was sufficient for this neuronal output^13^.

### Minimum Input Nodes to Control Network

**Figure 3:**
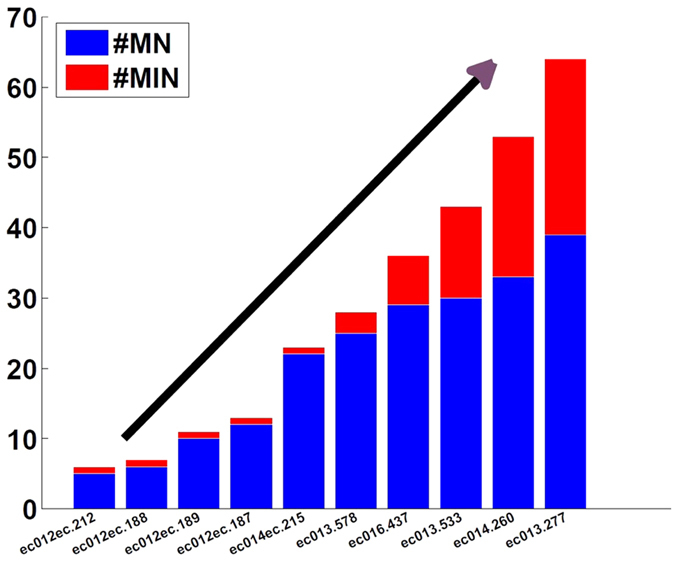
Minimum Input Nodes for 10 sessions. #MN is the number of matching nodes in the reconstructed network, #MIN is the number of minimum input nodes to control the whole network. #MN + #MIN is the total number of neurons involved in the reconstructed network.

As shown in Fig. 3, when the size of the reconstructed network is small, one can find a perfect matching in the directed graph, which means only one node in the network can control the dynamics of the whole network. As more neurons become involved in the network, more input nodes (*driver nodes*) need to be controlled. More complex network require more driver nodes to control the whole network in entorhinal-hippocampal regions of rats.

### Simulating the Topology of a Biological Neural Network

The actual synaptic connections between two neurons were shown to depend on their physical distance^9^,^14^, which leads us to use two distance-dependent models to simulate a neural network and make comparisons between them. Our results show that this idea can help form small-world properties very well: (1) small average path lengths and (2) large average clustering coefficients, which meet with experimental data and our interest in analyzing the small-world features of the sampled network compared to the original unknown network. *Model #1* is


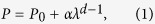


while *model #2* is


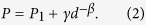


**Figure 4:**
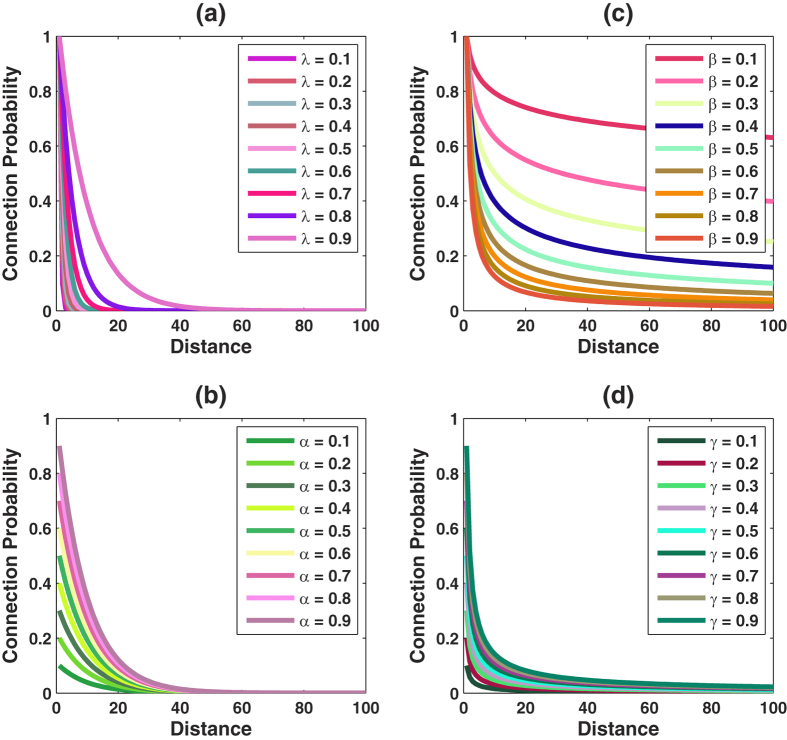
Connection probability for two distance-dependent models with different *λ*, *β*, *α*, *γ*. (**a**,**b**) show the performance of connection probability with different *λ* or *α* of model #1; (**c**,**d**) show the performance of connection probability with different *β* or *γ* of model #2.

Here, *d* is the distance between two neurons. The parameters *α* and *λ* are in (0, 1). If *α* is 1, the neighbours are certainly connected by edges with length 1. If *α* < 1, the global connection depends on both parameters as shown in Fig. 4(a,b). As *λ* increases from zero, the expected number of long-range associations increases and *α* determines the initial states of the probability of connections among neighbors, with negligible influence on the longest range to establish connections.

One significant difference between these two models is the final state, which is the probability of connection at longer range. Figure 4(c,d) shows the effect of varying the parameters of *model #2*. We set *γ* equals to 1. When *β* increases, the final state is decreasing but it is larger than 0. *Model #2* provides a condition when the range between two neurons is long, they can form a connection with a higher probability when compared with *model #1*. *Model #2* works to change the initial states of connections by adjusting *γ* and gives a big tolerance to a long-range connection formation. The biases *P*_0_ and *P*_1_ in these two models are used to adjust the performance of the outputs, and without loss of generality, we set them to 0 in the following discussion.

We simulated a biological neural network based on the above distance-dependent probability models in a three-dimensional space, which reflects the actual relationship better than one-dimensional or two-dimensional grids that neglected unique spatial properties of entorhinal-hippocampal structure. Although one or two-dimensional grids is convenient mathematical derivation of graph properties, intensive simulations of three-dimensional model would reveal more realistic graph properties of biological neural networks. Six electrodes were randomly inserted into this space to measure neurons extracelluarly. The number of electrodes inserted in the experimental datasets is different across the sessions. An average number of electrodes (*six*) was included in the simulations. Only neurons close to the electrodes would be sampled.

### Performance of Two Metrics for Describing Small-world-ness

**Figure 5:**
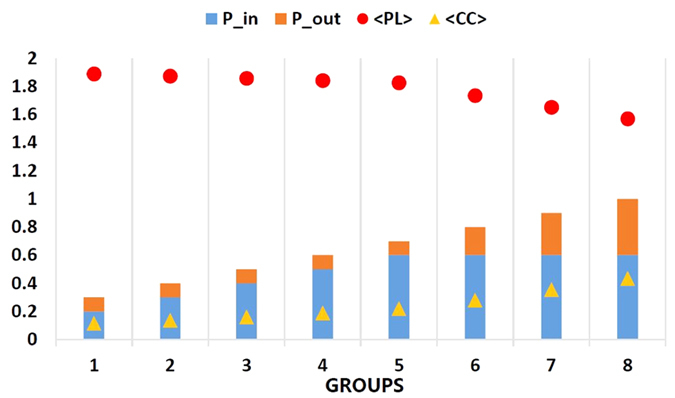
Performance of distance-dependent model. 8 groups were explored to analyze different combinations of *P*_*in*_ and *P*_*out*_ influencing 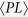, 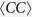 respectively.

The distance-dependent probability *model #1* and *#2* both indicate that the longer distance between two neurons lowers the connection probability. In each simulation, 500 randomly distributed neurons were generated as test nodes. A distance threshold *l* was set. When the distance between two neurons is larger than *l*, they have a probability *P*_*out*_ to form a connection; otherwise, they are connected with a probability *P*_*in*_. Normally, *P*_*in*_ > *P*_*out*_. Figure 5 shows the influence of different sets of *P*_*in*_ and *P*_*out*_ on 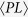 and 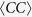. When the sum of these probabilities are larger, 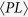 is smaller and 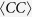 is higher. Note that when *P*_*in*_ is increased in the first 5 groups, the slopes of 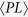 and 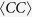 are smaller than that when increasing *P*_*out*_ in the last 3 groups. Then a linear model is used to fit 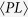 and 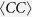 using both *P*_*in*_ and *P*_*out*_ respectively. The results could be described by


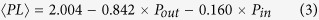


and


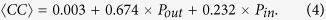


Here, *R*^2^ are 0.9994 and 0.9897 respectively, which show that 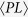 and 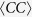 have strong linear relationships with the two connection probabilities, *P*_*in*_ and *P*_*out*_. We have simulated 50 random graphs adjusting the local region of *P*_*in*_, which can accurately represent the 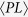 and 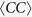 of those derived from experimental data as summarized in Table 1. We used genetic algorithm to perform global optimization to minimize the differences of patterns (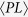 and 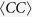) between experimental and simulated networks, which does not rely on the initial values of *P*_*in*_ and *P*_*out*_. As shown in Table 2, our model is sufficient for providing us with similar properties as experimental data, especially when the size of network is large (N ≈ 50).

**Figure 6:**
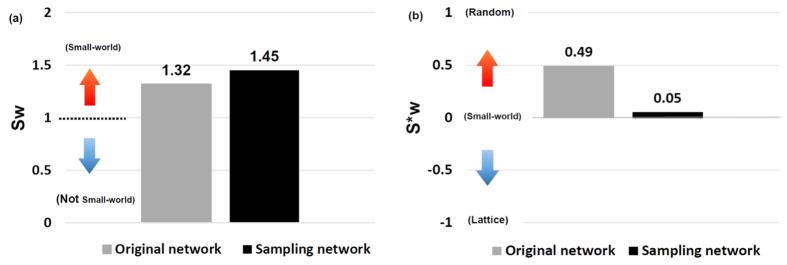
Small-world-ness of the original and the sampled networks described by two metrics. (**a**) *S*_*w*_ larger than ‘1’ indicates it is a small-world network; (**b**) 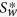 close to ‘0’ indicates it is a small-world network, close to ‘1’ or ‘−1’ presents it is more like a random or lattice network.

Without loss of generality, we selected *P*_*in*_ = 0.6 and *P*_*out*_ = 0.1 to analyze the performance of the two small-world-ness metrics. As shown in Fig. 6, the metrics *S*_*w*_ of *ON* and *SN* are both larger than 1, which indicates that *ON* and *SN* are small-world networks. *S*_*w*_ of *ON* is significantly different from the base value 1, with *P-value* (P) of *t-test* close to 0, lower than the significance level 0.05. For *S*_*w*_ of *SN*, compared with base value 1, P ≈ 0, and compared with *S*_*w*_ of *ON*, P = 0.00014. For 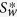 of *ON*, compared with base value 0, P = 0. When compared with base value 0, P = 0.05 for 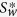 of *SN*, and compared with 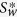 of *ON*, P ≈ 0. Yet, the small-world-ness is overestimated. The metric 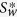 provides more information about the network properties. When 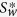 of *ON* is 0.49, it indicates that *ON* is a weak small-world network. Actually, the *S*_*w*_ of *ON* is 1.32, slightly larger than 1, so we can not consider it as a strong small-world network. However, *S*_*w*_ can not convey information clearly as there is no limit on the values taken by *S*_*w*_. The metric 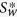 of *SN* is 0.05, so it is more like a small-world network as compared with *ON*. This case shows that, based on the distance-dependent probability model, 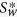 conveys much more information, and can distinguish the properties of different networks clearly.

**Figure 7:**
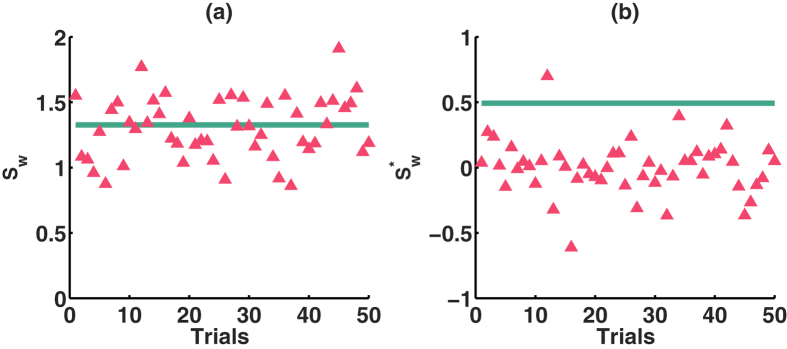
Differences of the small-world-ness between the original and the sampled networks. Details of 50 trials with different multi-electrode positions. Green lines indicate the small-world-ness of *ON*, and red triangles are that of *SN*. (**a**,**b**) show the performance of *S*_*w*_ and 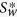.

The values displayed in Fig. 7 are the calculated values of averaging 50 realizations with the 6 electrodes randomly inserted in a three-dimensional space. Then, we found that the standard deviance of *S*_*w*_ and 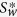 for *ON* is 0.0013 and 0.0005, while the *SN* is 0.63 and 0.25, respectively, indicating that *S*_*w*_ is very sensitive to the positions of electrodes when the network size is small. In real experiments, the electrodes are inserted without much prior knowledge to aid the estimation of the small-world-ness, so a consistent metric is preferred to describe this property and then justify whether we overestimate or underestimate the small-world features. In Fig. 7, we list the details of 50 trials, which show the differences of small-world-ness for *SN* and *ON*. *S*_*w*_ varies a lot in different trails. However, the performance of 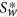 is consistent over 50 trials. It shows that we consistently overestimated the small-world-ness when using *SN* except for one case.

**Figure 8:**
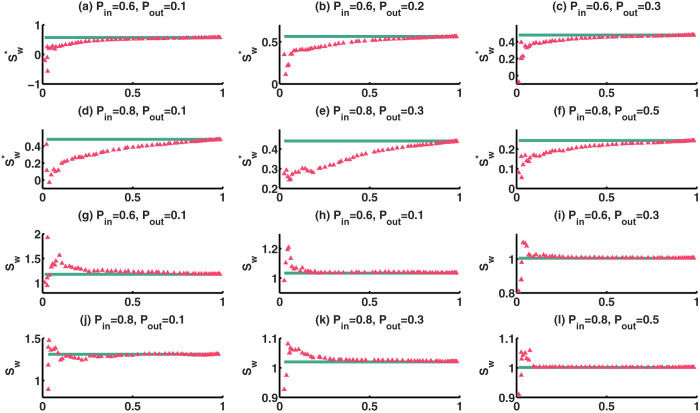
Performances of the two small-world-ness metrics with increasing sampling percentage and several combinations of two connection probabilities. Green lines indicate the small-world-ness of *ON*, and red triangles are that of *SN*.

In reality, only the neurons distributed around each electrode can be sampled in experiments. Hence, the ability of each electrode to detect electrical signal determines the number of sampled neurons. We simulated different *P*_*in*_ and *P*_*out*_ connection probabilities to see how the two small-world-ness metrics are changed with the increase in the percentage of sampled neurons. In the three-dimensional space, we inserted six electrodes to sample neurons around them. We set a threshold *l*′, and only the neurons distributed within the range *l*′ of the electrode can be sampled. By enlarging this threshold, more neurons can be recorded. Figure 8, (*a*) − ( *f*  ) suggest that, when the percentage of sampled neurons is increasing, the 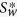 of *SN* is always smaller than that of *ON*. This demonstrates that 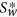 overestimates the small-world-ness of *ON* based on *SN*. Figure 8, (*g*) − (*l*) indicate that when the percentage of sampled neurons is below 20% (*100* *neurons*), the *S*_*w*_ of *SN* is smaller than that of *ON*, but it would quickly increase to the peak, giving an indication of overestimating the small-world-ness. However, when the number of sampled neurons increases, the *S*_*w*_ of *SN* is close to that of *ON*. Figure 8 also shows that 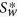 can give a consistent overestimation in the small-world-ness feature from *SN*. However, it converges to the small-world-ness of *ON* with higher sampling percentage. On the other hand, *S*_*w*_ is unable to provide us with a consistent result when the number of sampled neurons is small. Yet, *S*_*w*_ converges to the actual small-world-ness more quickly than 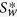.

### Estimation of Actual Small-world-ness Based on Sampled Topology

**Figure 9:**
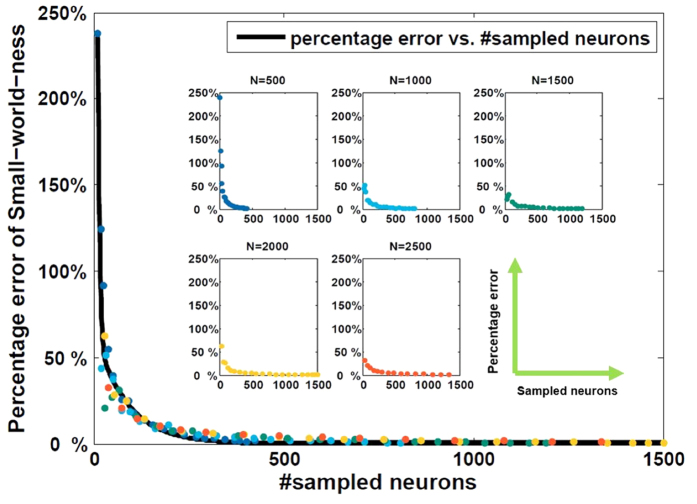
Percentage error of small-world-ness estimated from sampled networks. Different combinations of the size of networks were analyzed.

As the 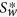 gives us a consistent and promising method to describe the small world feature of the network in multi-electrode sampling case, we use it as a quantitative method to evaluate the small-world-ness. To mimic experimental situation, we should estimate the number of neurons in the measured region. The region is approximate to a cuboid. The overall average density of the brain is around 9.2 × 10^4^ *neurons*/*mm*^3^ and 7.2 × 10^8^ *synapses*/*mm*^3 ^15. The edge density is therefore around 8.5%. Here we have 4 shanks with intershank distance around 200 *μ*m, and the electrodes measured approximately a column in 50 *μ*m radius. Thus, we can calculate the length of the piece of tissue measured to be around 780 *μ*m, the width is approximately 320 *μ*m, and the height of tissue measured is around 100 *μ*m. The approximate number of neurons is around 2296. If a tighter tissue measurement is adopted, less neurons will be included in the regions measured. We simulated different sizes of networks (*N* = *500*, *1000*, *1500*, *2000*, *2500*) with various combinations of *P*_*in*_ and *P*_*out*_, and then sampled different numbers of neurons from networks to find the relationship between the small-world-ness of the original network and that of the sampled network. The distance-dependent model configuration is straightforward to simulate networks with similar properties as biological networks, such as edge density, and is also convenient to form small world networks. The combination of *P*_*in*_ and *P*_*out*_ ensures that we can simulate a variety of edge density of brain network. We take *P*_*in*_ = 0.25 and *P*_*out*_ = 0.05 to get the edge density about 8.32% (≈8.5%) as an example in Fig. 9.

The percentage error (*σ*) of our simulated network is calculated by the small-world-ness of original network 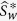 and sampled network 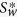 using


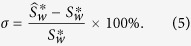


In Fig. 9, we intend to find the relationship between percentage error and the number of sampled neurons no matter what the size of original network is. Two-term exponential function was selected to fit our simulated data. The general model of our fitted function is


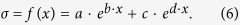


Coefficients of the equation are a = 1.564, b = −0.080, c = 0.279, d = −0.006, within 95% confidence bounds. Root mean squared error (*RMSE*) is 0.060, and *R*^2^ is 0.8494, which indicate that the fitted model can capture the relationship between the percentage error and the number of sampled neurons. Finally, we can make an adjustment to the previous result to infer the true small-world-ness 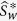 of original network as following


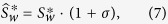



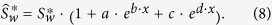


Substitute equation (21) into equation (8), we can get the final model to calculate the actual small-world-ness,


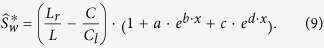


Thus, 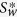 of sampled network could be adjusted by equation (9) to infer actual small-world-ness. By exploring edge density probabilities from 5.8% to 23%, which is consistent with estimation of cortical connectivity from pair-wise cell recordings^16^, the corresponding range of adjustment can be identified for reference.

In Table 3, the results are adjusted accordingly, and the small-world networks identified remain to be significant.

## Discussion

The reconstructed networks in this study show that the neural network in the entorhinal-hippocampal region of well-trained rats demonstrate small-world features, which are consistent with some previous studies^17^,^18^. Such small-world network has been observed not only at the regional level but also at the neuronal level^16^,^18^. However, few previous studies have been conducted on constructing networks based on multi-neuron recordings of local circuits during behavioral tasks. We focused on the entorhinal-hippocampal region at the level of cells when a rat was performing a task for several rats with different tasks. Our results on small-world properties of networks reconstructed from individual neurons are consistent with previous studies^17^. However, while their methods are suitable for reconstructing small networks, sparse *GLM* is more desirable when estimating larger numbers of parameters if more neurons are involved in the network. Furthermore, complex network studies, which investigate whether the properties derived from the sampled network could accurately represent the original network accurately at the neuronal level, are lacking. We have, therefore, compared two popular metrics estimating small-world-ness based on a multi-electrode sampled network. Finally, we estimated the true small-world-ness of the original unknown network based on the number of neurons recorded. Our results also show that, when the network size is small, it can be controlled with a few nodes, where these networks are homogeneous and dense. When the network size is increasing, it becomes much more difficult to control the whole network.

We have also compared the similarities and the differences of neural networks at different training stages. As shown in Table 1, *Rat #1* did the same task *Mwheel* for 10 times, the size of reconstructed network is similar. Although the placement of electrodes and training time are the same for *Rat #2* for example, we found that for different tasks, the size of the reconstructed network is quite different. Furthermore, when the training time increases for the same task like *Rat #4*, more neurons and more connections are involved in the functional network, which is in agreement with previous studies^19^. The results show us that when the rats conducted different tasks, their brain functional networks showed significant small-world features. However, different tasks or different training stages influenced the size of reconstructed network. With the further development of statistical models for network reconstructions in the future, we hope to be able to understand more about functional anatomy of cell assemblies. Instead of thresholding a binary matrix as in our case, the model should work directly with both directed and weighted networks. Our sparse kernel *GLM* to reconstruct functional connectivity provides a topology for analysis of other network properties such as the relationship between neuronal firing patterns and community modules. When the size of a reconstructed neural network increases, detecting communities can provide valuable information for understanding biological systems^20^.

We have observed that the graph theory has been commonly used to analyze large-scale networks such as different brain regions in neuroscience, but very few studies analyzed networks at the neuronal level. Also, only limited portion of neurons could be recorded by multi-electrode techniques. When the original network is sparsely sampled, the average path lengths and average clustering coefficients are quite different from that of the actual network. Therefore, we have utilized several quantitative methods to evaluate the small-world-ness. In this paper, we test the performance of two commonly used small-world-ness metrics based on a widely used subsampling scheme. After intensive simulations based on the distance-dependent probability model, a quantifiable relationship between the error in the estimated small-world-ness and the number of sampled neurons has been identified. Along the way, we have found possible adjustments to the small-world-ness measures to reflect the actual network properties from sampled data. Such rules were then applied to adjust the small-world-ness measures of the reconstructed microcircuit network in the earlier analysis of experimental data. In the range around average edge density observed in the brain, the corrected measures indicate the *EC-CA1* circuitry is indeed a small-world network. Our results show that one can use the number of sampled neurons to estimate a more accurate small-world-ness compared to that of sub-graph, which could be applied to the studies of other small-world networks when there are only sampled data available.

## Methods

### Experimental Data

The datasets, curated from *CRCNS*^1^, contain multi-unit recordings from the entorhinal-hippocampal regions of 11 rats when the animals were conducting different behavioral tasks. The data was obtained from 442 recording sessions, and in each session the animal finished one of 14 behavioral tasks. The details of analyzed sessions are shown in Table 4. The selected sessions contain 4 different rats doing 4 tasks and the number of recorded neurons number ranges from 25 to 105. The neurons were recorded from 5 different brain regions. The selected sessions can help us do comparative studies. The neurons with firing rates less than 0.5 Hz and isolated ones in final reconstructed network are left out.

### Generalized Linear Model

Spike trains can be viewed as point processes^21^. The conditional intensity function 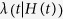 based on the firing probability *H*(*t*) in the past can generate an instantaneous firing rate, which can be explained as


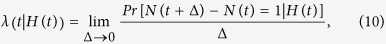


where *N*(*t*) denotes the number of events which occurred within the time interval [0, *t*]^22^. We take each neuron’s spiking activity as the output, and the spike trains from all the other neurons as the input, to establish a *GLM*. The estimated coefficients can be regarded as coupling strengths between the output neuron and input neurons. One can estimate the coefficients using the *GLM* as


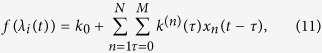


where *k*_0_ is a scalar zeroth-order kernel function, and *k*^(*n*)^ are first-order kernel functions describing the relationship between the output neuron *i*.’s spike probability *λ*_*i*_(*t*) and the *n*-th input *x*_*n*_(*t*). Without loss of generality, the first-order model will be adopted for demonstration in this session. Here, is a known link function. Since the firing rate of neuron *i* obeys a Gaussian distribution, the equation (11) can be written as


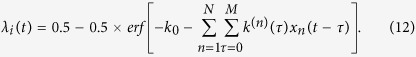


### Global Basis Functions

From equation (11), it can be seen that the number of parameters to estimate is *N* × (*M* + 1) + 1. Thus, if a long memory of the neuron is taken into consideration, meaning *M* is large, too many estimated parameters need much computational efficiency. A common method to reduce the number of model coefficients is the functional expansion technique, which first decomposes the kernel functions *k*^(*n*)^(*τ*) into


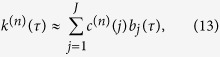


where 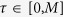. Substituting equation (13) into (11), one gets


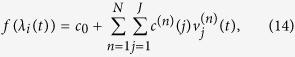


where 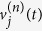 is the convolution of the *j*-th basis function and the *n*-th input:


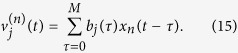


The number of model coefficients have reduced to *N* × *J*, and we can control the order of the basis function *J* to reduce the model complexity. Furthermore, in order to reduce the complexity in estimating *b*_*j*_(*τ*), one can use a global basis to span the entire system memory, which can take place on *b*_*j*_(*τ*). In this case, *b*_*j*_(*τ*) is represented as a Laguerre basis function^23^, which has been previously used in modeling physiological systems^24^. The *j*-th order Laguerre basis function *b*_*j*_(*τ*) constitutes an orthonormal basis, and 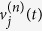 can be calculated recursively^25^, as


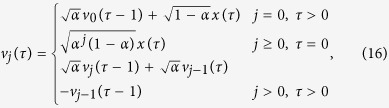


where *α* is the Laguerre parameter which determines the rate of decay of the kernel functions. The smaller the *α* is, the faster the *v*_*j*_(*τ*) function decays. Laguerre basis functions with optimal *α* reduce the number of coefficients to be estimated. Thus, the structure of the *GLM* can be reconstructed from these functions efficiently.

### Sparse Generalized Linear Model

Lasso regularization aims to minimize the sum of squared errors by adding a constraint on the sum of absolute values of the parameters to be estimated. It ensures that only the variables significant to the prediction of the output spike trains are selected in the model, thus, avoids overfitting.

Applying the Lasso method to equation (14) results in


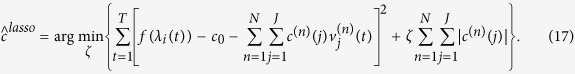


A wide range of *ζ* from 10^−5^ to 10^−1^ was explored. For each *ζ*, deviance was obtained from 5-fold cross-validation. The *ζ* with smallest deviance and the corresponding set of 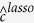 were selected as the estimated coefficients.

### Model Validation

The model described in this study can provide the conditional probability of the output spikes. The goodness-of-fit was tested with time-rescaled Kolmogorov-Smirnov (*K-S*) test^26^. If the model is accurate, it should provide a conditional firing rate function changing the recorded spike-train data into a Poisson process, and the plot of the transformed values should be independent and distributed around the 45-degree line on the K-S plot. The dependencies between subsequent intervals were inspected using the autocorrelation method.

### Graph Analysis

The network established can be described using graph theory. We aim to find the basic and essential characteristics of the nodes, edges, and topological structure of the neural circuits. The resulting mathematical models will be useful to describe and predict the dynamic behavior of the network. The reconstructed network is described by three key measures^27^:

#### Degree and Degree Distribution

The degree of one node is defined by the number of edges connected to it. The node degrees existing in the network is described by a distribution function *P*(*k*), which is the percentage of the nodes with degree *k* among the total number of nodes.

#### Average Path Length

The path length *d*_*i*,*j*_ is the number of edges existing in the shortest path connecting node *i* and *j*. Average path length (*L*) is the average over the sum of all *d*_*i*,*j*_. Small *L* is one of the small-world features.

#### Clustering Coefficients

The immediate neighbors of one node can form a cluster. This measure shows the actual ratio of the number of connections among the neighbors and the maximum number of connections among them. A large average clustering coefficient over the whole network is also a small-world feature.

### Control of Reconstructed Neural Network

The reconstructed network in this study can be regarded as a directed graph. After obtaining the reconstructed network from datasets, we are interested in finding the “*key*” neurons which can take charge of the whole network. In 1970s, Lin *et al*. proposed the idea of structural controllability^28^ based on the linear time-invariant (*LTI*) systems. In 2011, Liu *et al*.^11^ studied directed networks based on structural controllability. The fundamental equation of a LTI system is:


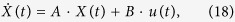


where 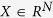 is the state vector, 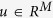 is the input vector, 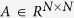 is the state matrix, and 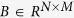 is the input matrix. The element *a*_*ij*_ in the state matrix *A* is 0, if there is no link from node *j* to node *i*. Because the parameters in both A and B matrices are either independent free ones or zero, A and B are called structured matrices. The structured properties show that in a real system, if one can fix the parameters to some values so that the system is controllable, then the system is *structurally controllable*.

In Liu *et al*.^11^, it is proved that in order to fully control a network of size *N*, the minimum number of input vertices (*N*_*i*_) needed is related to the size of maximum matching (M^*^) in the corresponding digraph:


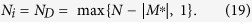


If the directed graph has a perfect matching, the minimum input vertice is 1; otherwise, it is equal to 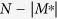.

In an undirected graph, a matching *M* is an independent edge set without common vertices. In a directed graph, an edge subset *M* is a matching if none of the edges therein share a common starting vertex or a common ending vertex. We find the maximum matching of a directed graph by transforming it into an undirected bipartite graph. The Hopcroft-Karp algorithm is chosen to determine the maximal matching in the corresponding transformed bipartite graph as this algorithm is both accurate and efficient for the small size neuronal network commonly recorded (<100 neurons).

### Approaches to Describe Small-world-ness

Our study shows that the reconstructed functional neural network presents significant small-world features due to its large average clustering coefficient 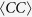 and small average path length 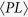. Although these two parameters have been widely adopted to identify a small-world network^29^, they were not defined for comparing the small-world-ness among different networks. For example, network A has larger 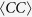 but also larger 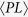 than network B, so we can not tell which network shows stronger small-world-ness. Thus, it is necessary to use a quantitative measure to describe the small-world feature. In an earlier study, the significance in small-world-ness of a network could be described by the following metric^30^


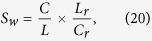


where *C*_*r*_ and *L*_*r*_ are 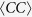 and 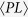 from the random graph with the same number of nodes and edges as that of the reconstructed network. A small-world network usually has 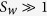, which should meet 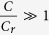 and 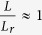. However, this metric is sensitive to the size of network, and it becomes larger as the size of network increases. These drawbacks in metric *S*_*w*_ have been tackled by another metric to describe the small-world-ness^31^. It focuses on analyzing whether the measured network is more like a regular lattice or a random graph. The Watts and Strogatz model was proposed to produce a small-world network which had both large 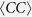 as with the corresponding regular lattice and small 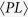 as with the random graph. The new metric is based on these two properties, and the new measure is


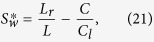


where *C*_*l*_ is the 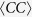 from the corresponding regular lattice. When the new metric is close to 0, the network shows stronger small-world-ness feature, which means that *C* ≈ *C*_*l*_ and *L* ≈ *L*_*r*_. When 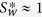, the network is more like a random graph. Whereas, when 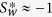, it has characteristics like a regular lattice. This metric is not sensitive to the size of network. We consider both these two metrics for measuring the small-world-ness of the network model.

## Additional Information

**How to cite this article**: She, Q. *et al*. Evaluating the Small-World-Ness of a Sampled Network: Functional Connectivity of Entorhinal-Hippocampal Circuitry. *Sci. Rep*. **6**, 21468; doi: 10.1038/srep21468 (2016).

## Figures and Tables

**Table 1:** Accuracy of prediction and properties of the reconstructed functional network for 10 sessions.

Session ID	Task	#Training	#Neurons	Regions	TP	TN	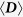 *	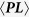 ^**^	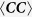 ^***^
ec012ec.187(Rat #1)	Mwheel	10	13	EC3 EC5	75.5%	92.1%	9.38	1.22	0.81
ec012ec.188(Rat #1)	Mwheel	10	7	EC3 EC5	73.3%	90.9%	5.71	1.05	0.95
ec012ec.189(Rat #1)	Mwheel	10	11	EC3 EC5	76.5%	92.8%	8.73	1.12	0.89
ec012ec.212(Rat #1)	Mwheel	10	6	EC3 EC5	84.9%	80.8%	2.00	1.87	0.39
ec013.533(Rat #2)	Linear	10	43	EC3 EC4 EC5 CA1	74.2%	90.3%	20.8	1.51	0.57
ec013.578(Rat #2)	Wheel	10	28	EC3 EC4 EC5 CA1	74.9%	88.9%	20.9	1.22	0.79
ec016.437(Rat #3)	Wheel	8	36	EC3 EC4 EC5 CA1	79.2%	80.3%	14.8	1.58	0.50
ec014.215(Rat #4)	Bigsquare	1	25	EC2 EC3 EC5 CA1	80.1%	92.0%	20.6	1.21	0.79
ec014.260(Rat #4)	Bigsquare	3	53	EC2 EC3 EC5 CA1	80.0%	93.1%	38.2	1.26	0.74
ec014.277(Rat #4)	Bigsquare	4	64	EC2 EC3 EC5 CA1	84.2%	95.0%	46.9	1.25	0.76

*Average Degree.

**Average Path Length.

***Average Clustering Coefficient.

**Table 2:** Simulated small world patterns.

Case	Experimental Data		Our Model	
	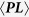	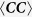	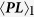	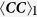
ec012ec.187	1.22	0.81	1.31	0.75
ec012ec.188	1.05	0.95	1.21	0.97
ec012ec.189	1.12	0.89	1.14	0.97
ec012ec.212	1.87	0.39	1.89	0.41
ec013.533	1.51	0.57	1.49	0.54
ec013.578	1.22	0.79	1.20	0.83
ec016.437	1.58	0.5	1.60	0.45
ec014.215	1.21	0.79	1.22	0.82
ec014.260	1.26	0.74	1.25	0.76
ec014.277	1.25	0.76	1.25	0.77

**Table 3:** Small-world-ness adjustment on 10 sessions.

Session ID	#Neurons	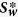	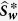
ec012ec.187(Rat #1)	13	0.0201	[0.0286 0.0365]
ec012ec.188(Rat #1)	7	0	0
ec012ec.189(Rat #1)	11	0.0038	[0.0059 0.0086]
ec012ec.212(Rat #1)	6	−0.0357	[−0.2887 0.0799]
ec013.533(Rat #2)	43	0.1716	[0.2174 0.2422]
ec013.578(Rat #2)	28	0.0352	[0.0475 0.0500]
ec016.437(Rat #3)	36	0.2288	[0.3005 0.3377]
ec014.215(Rat #4)	25	0.1020	[0.1384 0.1621]
ec014.260(Rat #4)	53	0.0451	[0.0553 0.0603]
ec014.277(Rat #4)	64	0.0429	[0.0515 0.0565]

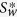
 Small-world-ness of sampled network.

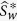
 Small-world-ness of original network when the edge density is from 5.8% to 23%.

**Table 4:** Number of neuronal units recorded in 10 experiment sessions.

Session ID	*N*_0_*	N**	Percentage	Regions***	Task
ec012ec.187(*Rat #1*)	25	13	52%	EC3 EC5	Mwheel
ec012ec.188(*Rat #1*)	25	7	28%	EC3 EC5	Mwheel
ec012ec.189(*Rat #1*)	25	11	44%	EC3 EC5	Mwheel
ec012ec.212(*Rat #1*)	20	6	30%	EC3 EC5	Mwheel
ec013.533(*Rat #2*)	85	43	51%	EC3 EC4 EC5 CA1	Linear
ec013.578(*Rat #2*)	87	28	32%	EC3 EC4 EC5 CA1	Wheel
ec016.437(*Rat #3*)	75	36	48%	EC3 EC4 EC5 CA1	Wheel
ec014.215(*Rat #4*)	124	25	20%	EC2 EC3 EC5 CA1	Bigsquare
ec014.260(*Rat #4*)	75	53	71%	EC2 EC3 EC5 CA1	Bigsquare
ec014.277(*Rat #4*)	105	64	61%	EC2 EC3 EC5 CA1	Bigsquare

*The number of experimental recorded neurons.

**The number of neurons in reconstructed network.

***The recorded regions.
